# Targeted Therapy in Leukaemia, Lymphoma and Myeloma

**DOI:** 10.3390/jpm12010074

**Published:** 2022-01-08

**Authors:** Stephen Samuel Opat

**Affiliations:** School of Clinical Sciences at Monash Health, Monash University, Clayton, VIC 3088, Australia; stephen.opat@monash.edu

Historically, most advances in cancer therapy have been pioneered by clinicians managing the blood diseases. The panoply of illustrative examples includes the application of radiotherapy in Hodgkin disease, use of aminopterin to treat childhood lymphoblastic leukaemia, discovery of the Philadelphia chromosome, identification of the translocation in involving the ABL oncogene and targeting of ABL tyrosine kinase in CML, use of monoclonal antibodies to target B cell lymphoma and leukaemia, use of proteasome inhibitors in myeloma and development of drugs promoting apoptosis in leukemia and myeloma [1,2,3,4,5,6,7,8].

While many patients continue to benefit from standard cytotoxic chemotherapy, it is now apparent that further advances are unlikely to be realised with existing cytotoxic agents, and that progress will be based on our understanding of tumour biology and immunology.

There are now several therapies targeting specific genes and proteins involved in the growth and survival of cancer cells. This transformation is most apparent in lymphoma, leukaemia, and myeloma, where therapies targeting Bruton tyrosine kinase, phosphatidylinositol 3-kinase, B-cell lymphoma 2, the proteasome and the ubiquitin E3-ligase cereblon and are already in clinics [9,10,11,12,13].

The role of the immune system in tumour eradication has led to the development of second-generation CD20 antibodies, bi-specific antibodies and T-cell engagers, chimeric antigen receptor T-cells, and agents acting on key immune checkpoints such as programmed cell death protein 1 (PD1). Agents acting on DNA methylation or histone protein modification are also active in certain haemopoietic malignancies [14,15,16,17,18,19,20,21].

Progress in cancer is incremental, and despite many cancers sharing biological features, every tumour is unique. Loss of a single regulatory gene (e.g., TP53) can have a profound impact on the therapeutic response. While patients in general are physiologically similar, drug pharmacokinetics and pharmacodynamics are often heterogeneous with efficacy and tolerability impacted by age, comorbidity, prior therapies as well as drug–drug interaction.

This Special Issue of the *Journal of Personalized Medicine* entitled “Targeted therapy in Leukaemia, Lymphoma and Myeloma” contains 10 publications authored by experts working in a diverse range of haematological cancers including B cell non-Hodgkin lymphoma [22,23,24,25], T-cell non-Hodgkin lymphoma [26], Multiple Myeloma [27,28,29], Chronic Lymphocytic Leukaemia [23,25,28], Acute Myeloid Leukaemia [28,30] and Acute Lymphoblastic Leukemia [31].

These neoplasms are biologically distinct but share biological features which enable certain agents to be used across a broad range of tumours including BCL2 inhibitors in B cell non-Hodgkin lymphoma, Multiple Myeloma, Chronic Lymphocytic Leukaemia, Acute Myeloid Leukaemia [28,30]; hypomethylating agents in T cell Lymphoma and Acute Myeloid Leukaemia [26,30]; BTK inhibitors in Chronic Lymphocytic Leukaemia and B cell lymphoma [25]; Cereblon-Interacting Small Molecules in Follicular Lymphoma and Myeloma [22,27,29]; and Bispecific antibodies in B cell lymphoma and B Lymphoblastic leukaemia [24,31].

Use of monoclonal antibodies and their derivatives is a common theme across the spectrum of blood cancers with bare antibodies, antibody–drug conjugates and bispecific antibodies being active in Acute Myeloid and Lymphoblastic Leukaemia, B-cell and T-cell Lymphoma and Myeloma [22,24,26,29,30,31].

Several of these agents are proven to be superior to conventional chemoimmunotherapy but are not without risk. Tsai Y-F et al. provide a comprehensive review of hepatitis B virus reactivation in lymphoma patients receiving several of these agents [23]. The adverse event profile of many of these newer agents will take some time to define.

With the advancement in our understanding of tumour biology, many additional targets will be discovered; however, much of the future debate centres around sequencing of therapy, identification of rational combinations, the merits of fixed duration versus continuously administered therapy, long-term toxicity, and cost. 

The breadth of content in this issue makes it difficult to editorialise, with major paradigm-shifts in therapy for leukaemia, lymphoma and myeloma. Outcomes for patients with blood cancer appear to be improving, but much work stills needs to be done to identify optimal therapeutic strategies for various blood cancers in a diverse population of patients.

## Data Availability

Not applicable.
